# 

**Published:** 1915-09

**Authors:** 


### ANNOUNCEMENTS

MICHIGAN STATE BOARD OF DENTAL EXAMINERS. The next meeting of this Board will be held at Ann Arbor, beginning Monday, November 1st, at 8 A. M., and continuing through Saturday, November 6th. All dentists wishing to register for practice in Michigan should apply for full particulars to Dr. A. W. Haidle, Negaunee, Michigan.

EXAMINATION FOR POSITIONS IN THE U. S. ARMY DENTAL CORPS. Examinations will be held Monday, October 18th, at the following places: Ft. Slocum, N. Y.; Columbus, O., Barracks; Jefferson, Mo., Barracks; Ft. Logan, Colorado, and Ft. McDowell, Cal. For application blanks and full information applicants should write to the Surgeon-General, U. S. Army, Washington, D. C. Candidates must be graduates, between 21 and 27 years of age, of good moral character, and be citizens of the United States.

The compensation is $150 per month, traveling expenses, and certain army privileges. After three years of acceptable service there is a possibility of promotion t the rank and pay of a first lieutenant.

It is imperative that all applications shall be made at least two weeks previous to the examination.

